# Differences of muscle co-contraction of the ankle joint between young and elderly adults during dynamic postural control at different speeds

**DOI:** 10.1186/s40101-017-0149-3

**Published:** 2017-08-02

**Authors:** Yoshitaka Iwamoto, Makoto Takahashi, Koichi Shinkoda

**Affiliations:** 1 Graduate School of Biomedical and Health Sciences, Hiroshima University, 2-3 Kasumi 1-chome, Minami-ku, Hiroshima, 734-8553 Japan; 2 Department of Biomechanics, Graduate School of Biomedical and Health Sciences, Hiroshima University, 2-3 Kasumi 1-chome, Minami-ku, Hiroshima, 734-8553 Japan; 3 The Center for Advanced Practice and Research of Rehabilitation, Graduate School of Biomedical and Health Sciences, Hiroshima University, 2-3 Kasumi 1-chome, Minami-ku, Hiroshima, 734-8553 Japan

**Keywords:** Muscle co-contraction, Aging, Dynamic postural control, Electromyography, Performance speed

## Abstract

**Background:**

Agonist and antagonist muscle co-contractions during motor tasks are greater in the elderly than in young adults. During normal walking, muscle co-contraction increases with gait speed in young adults, but not in elderly adults. However, no study has compared the effects of speed on muscle co-contraction of the ankle joint during dynamic postural control in young and elderly adults. We compared muscle co-contractions of the ankle joint between young and elderly subjects during a functional stability boundary test at different speeds.

**Methods:**

Fifteen young adults and 16 community-dwelling elderly adults participated in this study. The task was functional stability boundary tests at different speeds (preferred and fast). Electromyographic evaluations of the tibialis anterior and soleus were recorded. The muscle co-contraction was evaluated using the co-contraction index (CI).

**Results:**

There were no statistically significant differences in the postural sway parameters between the two age groups. Elderly subjects showed larger CI in both speed conditions than did the young subjects. CI was higher in the fast speed condition than in the preferred speed condition in the young subjects, but there was no difference in the elderly subjects. Moreover, after dividing the analytical range into phases (acceleration and deceleration phases), the CI was larger in the deceleration phase than in the acceleration phase in both groups, except for the young subjects in the fast speed conditions.

**Conclusions:**

Our results showed a greater muscle co-contraction of the ankle joint during dynamic postural control in elderly subjects than in young subjects not only in the preferred speed condition but also in the fast speed condition. In addition, the young subjects showed increased muscle co-contraction in the fast speed condition compared with that in the preferred speed condition; however, the elderly subjects showed no significant difference in muscle co-contraction between the two speed conditions. This indicates that fast movements cause different influences on dynamic postural control in elderly people, particularly from the point of view of muscle activation. These findings highlight the differences in the speed effects on muscle co-contraction of the ankle joint during dynamic postural control between the two age groups.

## Background

Muscle co-contraction is the simultaneous contraction of agonist and antagonist muscles crossing a joint [1]. Fundamentally, in a single joint movement, an antagonist muscle is inhibited to allow an agonist muscle to work fluently; this is called reciprocal inhibition. During skilled movements, young individuals produce a net torque at the joint by activating agonist and antagonist muscles by optimally scaling [2]. Muscle co-contraction is essential for joint stabilization during refined motor performance.

Several studies showed a greater muscle co-contraction in elderly people than in young people [3–8]. The greater muscle co-contraction enabled elderly adults to control their postural sway well with stiffening their joint [9]. In contrast, Ge [10] described a rigid body movement induced by excessive muscle co-contraction that would potentially lead to a higher risk of instability upon postural disturbances. Moreover, strong muscle co-contraction increases the risk of excessive energy usage, resulting in fatigue [11]. Differing opinions exist regarding the biomechanical advantages/disadvantages of muscle co-contraction in elderly people. Examining the relationship between muscle co-contraction and postural sway in elderly people, Nagai et al. [4] showed a greater muscle co-contraction of the ankle joint during static standing, the functional reach test (FRT) [12], the functional stability boundary test (shifting body weight toward toes without heels off from quiet standing [13]), and gait in elderly people. They also showed a correlation between high muscle co-contraction and low postural control ability, while there was no relationship between muscle co-contraction and gait velocity. Hortobágyi et al. [7] compared antagonist leg muscle activation in young and elderly adults while walking at various speeds. Antagonist activation increased with gait speed in young adults but not in elderly adults. These findings suggest different patterns of muscle co-contraction adaptation, while increasing walking speed, in young and elderly adults. Elderly people may not be able to adapt to increasing gait velocity with appropriate muscle activation, compared to young people. Age-related speed deterioration was shown during normal walking [7] and dynamic postural control tasks [13]. We hypothesized that fast movements have different influences on dynamic postural control in elderly people in comparison with young people. This was particularly true for muscle activation. Moreover, the ability to rapidly recruit motor units may decrease with increasing age [14], whereupon elderly people begin to show postural movement slowing [15]. Poor postural control with muscle co-contraction is likely during fast dynamic postural control tasks in elderly people.

Therefore, it was important to evaluate the postural sway and the muscle co-contraction during dynamic postural control at fast speeds in elderly people. However, the effect of speed on muscle co-contraction of ankle joint during dynamic postural control among young and elderly people remains unknown. The objective of this study was to gain insight into muscle co-contraction effects on the ankle joint. We also sought to better understand mechanisms of dynamic postural control with muscle co-contraction in elderly people. We compared kinematic and kinetic characteristics and muscle co-contraction effects on the ankle joint in young and elderly adults during a functional stability boundary test at different speeds.

## Methods

### Participants

Fifteen healthy young adults (8 males and 7 females; mean age = 22.6 ± 1.4 years; mean height = 1.65 ± 0.08 m; mean body mass = 59.8 ± 11.4 kg) and 16 community-dwelling healthy elderly adults (7 males, 9 females; mean age = 73.2 ± 2.2 years; mean height = 1.56 ± 0.08 m; mean body mass = 57.2 ± 8.8 kg) participated in this study. We asked them to perform FRT [12]. In addition, for the elderly subjects, we asked them to answer the Modified Falls Efficacy Scale (MFES) [16] and fall history in the last 1 year. Subjects were excluded if they had dementia, neurological impairment, severe cardiovascular disease, persistent joint pain, or musculoskeletal impairment. Subjects gave written informed consent after receiving a detailed explanation of the purpose, potential benefits, and risks involved in the participation prior to this study. The experimental procedures used in this study were conducted in accordance with the Declaration of Helsinki and were approved by the Ethics Committee of the Division of Physical Therapy and Occupational Therapy Sciences, Graduate School of Health Sciences, Hiroshima University (No. 1401).

### Task

The task was a functional stability boundary test in the anterior direction. First, participants stood barefoot on two force plates (TF-400-A, Tec Gihan, Kyoto, Japan) in a natural position with heels separated at acromion interval distance. Their arms were crossed in front of their chest and subjects were required to gaze at a mark placed at eye level. Then, we asked them to shift their weight toward their toes, leaning forward as far as possible without lifting their heels from the force plates [13]. They performed the tasks in two speed conditions. In one condition, they moved at their preferred speed (preferred speed) and, in the other, as fast as they could (fast speed).

### Postural sway measurement

The kinematic data obtained during the functional stability boundary test were collected using Vicon MX, a three-dimensional motion analysis system (Vicon Motion Systems, Oxford, UK) with six infrared cameras. The kinetic data were collected by two force plates.

**Fig. 1:**
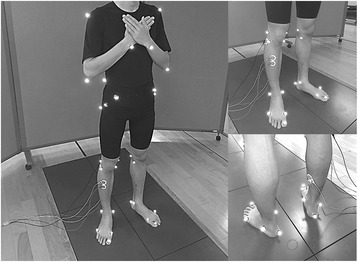
Data collection with all markers (*left*) and sensors (tibialis anterior: *right upper*, soleus: *right lower*) attached to a participant

Infrared-reflecting markers with a diameter of 14 mm were attached to 30 landmarks: one pair at the temple, lateral end of the superior nuchal line, tragus, acromion, olecranon, styloid process of the ulna, superior edge of the iliac crest, anterior superior iliac spine, posterior superior iliac spine, lateral and medial malleoli, heads of the first and fifth metatarsal, the tip of the toe, and the calcaneal tuberosity. Additionally, infrared-reflecting markers with a diameter of 9 mm were attached to 12 landmarks: a pair at the great trochanter, hip joint, lateral and medial epicondyles of the femur, and lateral and medial condyles of the tibia (Fig. 1). The spatial movements of the markers were captured by a three-dimensional motion analysis system at a sampling rate of 100 frames/s. At the same time, three-dimensional ground reaction forces were collected using the force plates at a sampling frequency of 2000 Hz.

The coordinates of joint centers were calculated according to methods described in previous studies [17, 18]. The ankle joint center was defined as the midpoint between the markers for the lateral and medial malleoli. The knee joint center was defined as the midpoint between the markers for the lateral and medial epicondyles of the femur. At first, for determining the hip joint center, a point one third of the distance between markers on the great trochanter and the anterior superior iliac spine was first determined bilaterally. Then, a line was drawn connecting these points. The points 18% medial to the ends of this line were defined as hip joint centers. The abdomen center was defined as the midpoint between the inferior edges of the lowest ribs. We constructed a rigid-body link model consisting of nine segments (thorax, abdomen, pelvis, both thighs, both shanks, and both feet) with the collected marker coordinates. We hypothesized that no energy was lost by deformity of the segments as well as conflict and compression of the joints in this rigid-body link model. Joint center coordinates and the location of the center of mass (COM) in each segment or the whole body were calculated using Bodybuilder software (Vicon Motion Systems, Oxford, UK). Data of marker coordinates, ground reaction force, body height and weight, and coefficients of each body segment inertia were recorded according to the method of Okada et al. [19].

**Fig. 2:**
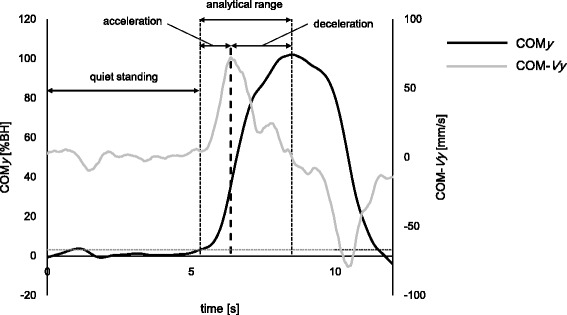
Representative example of COM*y* and COM-*Vy* during functional stability boundary test

An analytical range was determined by the displacement of the anterior-posterior COM (COM*y*; Fig. 2). The beginning of the range was defined as the moment when the COM*y* displacement was above its average ± 2 standard deviations in quiet standing. The end of the range was defined as COM*y*’s maximum (anterior: +).

We calculated the average peak anterior-posterior COP displacement (COP*y*) from the initial position (COP*y* excursion), the average peak COM*y* from the initial position (COM*y* excursion), and the mean and maximum velocity of COP*y* and COM*y* (COP-*Vy*_mean_, COP-*Vy*_max_, COM-*Vy*_mean_, COM-*Vy*_max_, respectively).

Additionally, we separated the analytical range into two parts with COM-*Vy*. One part was an acceleration phase when COM-*Vy* increased and the other was a deceleration phase when COM-*Vy* decreased (Fig. 2). The COP*y* and COM*y* for each subject was normalized individually to the subject’s body height (%BH).

### Muscle activation measurement

Electromyographic (EMG) data were collected using an EMG Master surface electromyogram (Mediarea Support Business Union, Okayama, Japan) at a sampling frequency of 2000 Hz. The skin of the dominant leg over the fibular head and the tibialis anterior (TA) and soleus (SOL) muscles was shaved and then cleaned with a skin pre-processing agent (Skin Pure, Nihon Kohden, Tokyo, Japan). Bipolar surface circular Ag/AgCl electrodes with a diameter of 34 mm (Blue-sensor M-00-S, Ambu, Ølstykke, Denmark) were placed every 30 mm in a center-to-center disposition in line with the muscle fibers [20] (Fig. 1). The ground electrode was affixed to the skin over the fibular head of the dominant leg.

EMG activity was recorded from the TA and SOL while the subjects were performing maximal voluntary contraction (MVC). The MVC of the TA was recorded during maximal isometric dorsiflexion of the ankle at 90° (anatomically neutral position), and the MVC of the SOL was obtained during maximal isometric plantar flexion. Strong verbal encouragement was given during every contraction to promote maximal effort.

The original raw EMG signal was band-pass filtered at a range of 20–500 Hz. We computed the root mean-square amplitude of the signal using a 50-ms window [4]. The EMG of each muscle was then expressed as a percentage of the EMG value during the MVC (%MVC). We calculated mean %MVC of TA and SOL to measure the muscle activity of them.

**Fig. 3:**
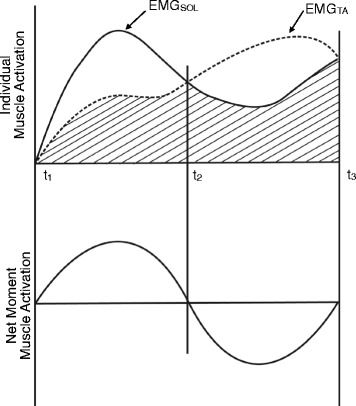
Hypothetical model of agonist and antagonist joint muscle activity to demonstrate the amount of co-contraction during movement (adapted from Falconer and Winter [21]). In this situation, from *t*_1_ to *t*_2_, TA was defined as an antagonist muscle and from *t*_2_ to *t*_3_ and SOL was defined as an antagonist muscle

To evaluate the relative level of co-contraction of the TA and SOL muscles, the co-contraction index (CI) was calculated using the method described by Falconer and Winter [21] (Fig. 3). Specifically, the following Eq. (1) was used:

1 $$ CI=\frac{2{I}_{\mathrm{ant}}}{I_{\mathrm{total}}}\times 100\% $$

where *I*_ant_ is the area of the total antagonistic activity, calculated using the following Eq. (2)

2 $$ {I}_{\mathrm{ant}}={\int}_{t_1}^{t_2}{EMG}_{TA}(t)dt+{\int}_{t_2}^{t_3}{EMG}_{\mathrm{SOL}}(t)dt $$

where *t*_1_ to *t*_2_ denote the period during which the TA EMG is less than the SOL EMG, *t*_2_ to *t*_3_ donate the period during which the SOL EMG is less than the TA EMG, and *I*_total_ is the integral of the sum of the TA and SOL EMGs while subjects performed the task, calculated using the following Eq. (3):

3 $$ {I}_{\mathrm{total}}={\int}_{t_1}^{t_3}\left[{EMG}_{\mathrm{agon}}+{EMG}_{\mathrm{ant}}\right](t)dt $$

We calculated CI during quiet standing, preferred speed condition (whole, acceleration, and deceleration), and fast speed condition (whole, acceleration, and deceleration) (Fig. 2).

### Statistical analysis

Data were analyzed using SPSS software (Windows version 22, IBM Japan, Tokyo, Japan). First, we applied the Shapiro–Wilk test to test all variables for normality. We set the statistical significant level at *p* = 0.05, and if the *p* value was lower than the significance level, we defined the variable as a non-normal data distribution. Then, if the data were distributed normally, we used Student’s *t* test. Otherwise, we used the Wilcoxon or Mann–Whitney test to compare the difference. We used a sequential Bonferroni correction to adjust the alpha level for multiple comparisons to counteract the problem of multiple comparisons [22].

## Results

After normality testing, COP-*Vy*_max_, mean %MVC of TA, and CI were defined as non-normally disturbed variables and are presented as medians (and interquartile range). The other variables were normally disturbed and are presented as mean ± standard deviation. On the FRT, data were missing for two young subjects and one elderly subject. The results of FRT, MFES, and fall history are presented in Table 1.

**Table 1:** The results of the functional reach teat test, Modified Falls Efficacy Scale, and fall history

	Young	Elderly
Functional reach test [cm]	35.46 ± 5.04	27.83 ± 5.13^*^
Modified Falls Efficacy Scale [score]	–	140
Fall history [frequency]	–	0

Data are presented as mean ± standard deviation

^*^Significant differences between the young and elderly groups (*p* < 0.01)

### Postural sway variables

There were no significant differences in COP*y* excursion and COM*y* excursion between the young and elderly subjects in both speed conditions (Table 2). Conversely, there were significant differences in velocity variables (COP-*Vy*_mean_, COP-*Vy*_max_, COM-*Vy*_mean_, COM-*Vy*_max_) between the two speed conditions (preferred and fast) in both age groups.

**Table 2:** Kinetic and kinematic variables of young and elderly subjects in terms of speed conditions

	Preferred speed		Fast speed	
	Young	Elderly	Young	Elderly
COP*y* excursion [%BH]	6.06 ± 1.01	5.63 ± 1.82	6.67 ± 0.84^†^	5.89 ± 1.65
	5.50–6.62	4.67–6.60	6.21–7.14	5.01–6.76
COM*y* excursion [%BH]	5.64 ± 1.05	5.11 ± 1.75	4.99 ± 0.98^†^	4.53 ± 1.61^†^
	5.06–6.22	4.18–6.03	4.45–5.53	3.68–5.38
COP-*Vy*_mean_ [mm/s]	2.30 ± 0.97	1.60 ± 0.63	5.18 ± 1.07^†^	4.14 ± 1.37^*†^
	1.77–2.84	1.26–1.94	4.59–5.77	3.42–4.86
COP-*Vy*_max_ [mm/s]	10.23 (7.94–11.80)	10.97 (9.17–13.01)	21.18 (18.41–23.45) ^†^	18.26 (14.17–19.94) ^†^
COM-*Vy*_mean_ [mm/s]	2.09 ± 0.80	1.44 ± 0.54^*^	3.66 ± 0.47^†^	3.06 ± 0.97^†^
	1.65–2.53	1.17–1.73	3.40–3.91	2.55–3.58
COM-*Vy*_max_ [mm/s]	4.86 ± 1.42	4.22 ± 1.32	7.39 ± 1.35^†^	6.04 ± 1.58^*†^
	4.08–5.64	3.52–4.91	6.65–8.14	5.20–6.88

Upper low: data are presented as mean ± standard deviation or median (interquartile range)

Lower low: 95% confidence interval

*COP* center of pressure, *COM* center of mass

^*^Significant differences between the young and elderly groups in each condition (*p* < 0.025)

^†^Significant differences between two conditions in each group (*p* < 0.025)

### Muscle activation

The elderly subjects had significantly greater mean %MVC values of TA and SOL muscles (*p* < 0.01, Table 3) in comparison with the young subjects.

**Table 3:** Mean normalized EMG activity (%MVC) in the analytical range during the functional stability boundary test

Condition	Muscle	Young	Elderly
Preferred speed	TA [%]	1.05 (0.94–1.27)	2.94 (1.75–5.30)^**^
	SOL [%]	21.49 ± 5.66	33.97 ± 13.18^**^
Fast speed	TA [%]	1.48 (1.10–2.23)	4.57 (2.98–9.47)^**^
	SOL [%]	21.42 ± 6.29	98 ± 11.87^**^

Data are presented as mean ± standard deviation or median (interquartile range)

*TA* tibialis anterior, *SOL* soleus

^**^Significant differences between the young and elderly groups (*p* < 0.01)

**Fig. 4:**
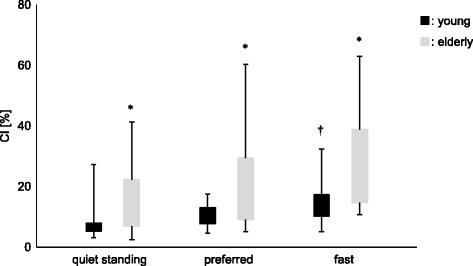
Co-contraction index (CI) between young subjects and elderly subjects during three different conditions (quiet standing, functional stability boundary with preferred speed, and functional stability boundary with fast speed). Significant differences (*p* < 0.05) between the young and elderly subjects are indicated by *significant differences (*p* < 0.025) between preferred speed and fast speed are indicated by †.

CI were significantly higher in elderly subjects than in young subjects for each evaluated condition (quiet standing, functional stability boundary test in the preferred speed and in the fast speed condition; *p* < 0.05; Fig. 4). In young subjects, the CI in the fast speed condition was significantly higher than in the preferred speed condition (*p* < 0.025). However, there was no significant difference between two speed conditions regarding the CI in elderly subjects.

**Fig. 5:**
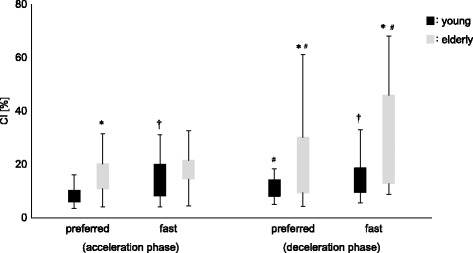
Co-contraction index (CI) between both age groups during functional stability boundary in each condition. The measures were also divided into two phases (acceleration, deceleration). Significant differences (*p* < 0.017) between ages (young, elderly) are indicated by *. Significant differences (*p* < 0.017) between conditions (preferred, fast) are indicated by †. Significant differences (*p* < 0.017) between phases (acceleration, deceleration) are indicated by #.

After dividing the analytical range in two phases (acceleration and deceleration), CI were significantly higher in elderly subjects than in young subjects, except for the acceleration phase in the fast speed condition. In young subjects, the CI was higher in the fast speed condition than in the preferred speed condition in each phase (acceleration and deceleration; *p* < 0.017). However, in elderly subjects, there were no significant differences between the two speed conditions in each phase. For elderly subjects, CI in the deceleration phase were significantly higher than in the acceleration phase in both speed conditions (*p* < 0.017, *p* < 0.025, respectively). In contrast, for young subjects, CIs were larger in the deceleration phase during the preferred speed condition only (*p* < 0.017; Fig. 5).

## Discussion

Previous studies reveal greater muscle co-contraction in elderly people during static and dynamic postural control, in comparison with young people [3–8]. However, no studies focus on the effects of speed of the muscle co-contraction of ankle joint during dynamic postural control, in young and elderly adults. We compared the differences of muscle co-contraction of ankle joint between young and elderly adults during a functional stability boundary test at different speeds. The present study addressed the following question: Do fast movements differently influence dynamic postural control in elderly people in comparison with that in young people, particularly regarding muscle activation? Three major findings were obtained. First, muscle co-contraction was larger in the elderly subjects than in the young subjects. Second, muscle co-contraction was higher in the fast speed condition than in the preferred speed condition in young subjects, but there was no difference in the muscle co-contraction between the two speed conditions in elderly subjects. Third, muscle co-contraction was higher in the deceleration phase than in the acceleration phase in both age groups (except for young subjects in the fast speed condition). To the best of our knowledge, this study is the first to compare the muscle co-contraction of the ankle joint between young and elderly subjects during dynamic postural control at different speeds.

In this study, there were no significant differences in the COP*y* and COM*y* excursions between the two age groups. Generally, both COP and COM are commonly used variables to evaluate postural sway [23, 24] and aging affects the movement of COP and COM during dynamic postural sway tasks [12, 13]. In the functional stability boundary test, older subjects with low risk of falls showed smaller COP amplitude in forward direction than young subjects [25]. This result is inconsistent with our results. However, in the current aging society, elderly people may present a great diversity of physical function. In this study, elderly subjects exhibited significantly shorter FRT distances than the young subjects. However, their average distance was similar to other studies that measured similar age groups, considering differences in body height [12, 26, 27]. Moreover, all elderly subjects demonstrated “140” on the MFES (full marks) and had no fall histories in the last 1 year. This indicated high falls efficacy without falls. Maki et al. [28] showed that subjects who reported fear of falling had worse COP measures during postural tasks. Tinetti and Powell [29] showed reduced falls efficacy was associated with declines in activities of daily living. Therefore, in this study, elderly subjects showed comparatively high ability to control their COP and COM during a functional stability boundary test.

Even though there were no statistically significant differences in the COP*y* and COM*y* excursions between both age groups in the different speed conditions, the CI of elderly subjects was greater than that of young subjects in both speed conditions. These results are consistent with a previous study measuring muscle co-contraction of ankle joint [4]. Examining individual muscle activity, both mean %MVC of TA and SOL were greater in elderly subjects than young subjects, during both speed conditions. Mean %MVC of TA in elderly subjects was about three to four times higher than in young subjects during both speed conditions. In contrast, for mean %MVC of SOL, elderly subjects showed only one and half times higher values in comparison with that of young subjects under both conditions. Lexell et al. [30] showed that reduced type 2 fiber size with increasing age and age-related reductions in muscle strength were caused by muscle atrophy. Johnson et al. [31] showed that the SOL consisted mostly of type 1 fibers and was not altered with age. Therefore, in this study, it was possible to increase EMG activity of TA compared with that of SOL in elderly subjects. As a result, our results showed larger muscle co-contraction of the ankle joint in elderly subjects compared to young subjects. In the previous study, subjects maintained their posture after moving their COM forward to measure the surface EMG [4, 5]. However, in this study, we measured the surface EMG to calculate CI during the movement of the COM. From the perspective of the COM, our results showed muscle activation in a more dynamic state of the COM. Woollacott and Tang [32] demonstrated the difference between static balance and dynamic balance. In this study, when the subjects performed the task, they did not move their base of support; thus, whether the functional stability boundary is dynamic or not is yet to be clarified. However, Duncan et al. [12] introduced the FRT, which is regarded as a test of dynamic balance and is very similar to our task. Considering the elderly participants in this study could move their COM and COP during the functional stability boundary test, greater muscle co-contraction of the ankle joint may be a new strategy for elderly people to control their posture without falling.

In contrast, the young subjects showed significant greater muscle co-contraction in the fast speed condition than in the preferred speed condition. However, elderly subjects did not show significant greater muscle co-contraction in the fast speed condition than in the preferred speed condition. Several research demonstrated that young people showed greater muscle co-contraction for more challenging tasks (e.g., walking in unstable shoes and running following isokinetic fatigue) [33, 34]. Moreover, Hortobágyi et al. [7] showed that young subjects had leg muscle co-contraction while walking with increased gait velocity. Performing the functional stability boundary test in anterior direction in fast speed (as fast as possible) would be a more challenging task, and thus, young subjects were able to increase leg muscle co-contraction with speed. As a result, young subjects may increase their muscle co-contraction at the ankle joint selectively for completing each task. Although the task at fast speed in this study could be more difficult for the elderly participants as well, they did not show significant increased muscle co-contraction of the ankle joint compared to that at the preferred speed. Additionally, their COP*y* excursion was not significantly different between the two speed conditions. Hof et al. [35] indicated that even if the COM is above the base of support, balance may be impossible if COM velocity is directed outward. Thus, it was necessary to move the COP at higher speeds, and young subjects showed such an adaptation. Our results showed that the elderly subjects may not be able to control the amount of muscle co-contraction depending on performance speed. Instead of increasing muscle co-contraction, the elderly subjects may stiffen their joints and lessen the movement toward a more challenging task.

We divided the analytical range into two phases (acceleration and deceleration phases) with COM-*Vy*. Additionally, we found some interesting results when calculating the CI in each phase and comparing the data. Elderly subjects showed significant greater muscle co-contraction in the deceleration phase than in the acceleration phase in both speed conditions. Tucker et al. [25] demonstrated that a rigid posture induced by a strong muscle co-contraction reduces the degrees of freedom in the postural control system. In the deceleration phase, subjects should prepare for stopping their COM from moving forward without elevating their heels from the ground and falling onto the ground. Hence, they increased the ankle joint stiffness with greater muscle co-contraction to maintain stability. However, at the same time, the excessive joint stiffness induced by the strong muscle co-contraction decreased the flexibility in postural control [25]. In this light, there is a possibility that stability is often disrupted when elderly individuals try to stop/decrease their movements in daily life. Young subjects increased muscle co-contraction in the deceleration phase during the preferred speed condition as well. However, they did not show greater muscle co-contraction in the deceleration phase than in the acceleration phase during the fast speed condition. Young subjects seemed to increase their leg muscle co-contraction selectively when they tried to move at fast speed, particularly in the acceleration phase. As a result, we did not find a difference between groups regarding each phase during the fast speed condition.

There were several limitations to our study. First, we did not measure the EMG of other lower extremity muscles, because our tasks focused on the ankle joint. Further studies are needed to clarify the relationship between the joint kinematics and muscle co-contraction with different muscles as a target. Second, the elderly participants in our study were community-dwelling and may have demonstrated relatively high levels of physical function. This may have affected between-group differences. However, elderly people demonstrate a wide variety of physical functioning [36, 37]; therefore, it was difficult to perform a comprehensive survey. Hence, we focused on pure age-related effects on postural control with muscle co-contraction of the ankle joint during functional stability boundary testing at different speeds. Third, the sample size was small, increasing the risk of type 2 statistical errors. However, we found several statistically significant between-group differences. Therefore, we believe that this limitation might not have a fatal problem in on our study.

## Conclusion

We compared postural sway parameters and muscle co-contraction of the ankle joint between young and elderly subjects during dynamic postural control at different speeds. Our results showed greater muscle co-contraction of the ankle joint, during dynamic postural control tasks in elderly subjects (in comparison with that in young subjects) during preferred and fast speeds. In addition, the young subjects showed increased muscle co-contraction during fast speeds compared with that in preferred speed. Elderly subjects showed no significant differences in co-contraction of the ankle joint muscles between the two speed conditions. This indicates that fast movements differently influenced dynamic postural control in elderly people, particularly with regard to muscle activation. Moreover, muscle co-contraction was larger in the deceleration phase than in the acceleration phase in the elderly subjects. These findings highlight the differences in the speed effects on muscle co-contraction of the ankle joint during dynamic postural control between the two age groups.

## Abbreviations

CI: Co-contraction index
COM: Center of mass
COM-*Vy*_max_: Max velocity of COM*y*
COM-*Vy*_mean_: Mean velocity of COM*y*
COM*y*: Anterior-posterior COM
COP: Center of pressure
COP-*Vy*_max_: Max velocity of COP*y*
COP-*Vy*_mean_: Mean velocity of COP*y*
COP*y*: Anterior-posterior COP
EMG: Electromyography
FRT: Functional reach test
MFES: Modified Falls Efficacy Scale
MVC: Maximum voluntary contraction
SOL: Soleus
TA: Tibialis anterior
