# Correction to: Differences of muscle co-contraction of the ankle joint between young and elderly adults during dynamic postural control at different speeds

**DOI:** 10.1186/s40101-017-0154-6

**Published:** 2017-11-15

**Authors:** Yoshitaka Iwamoto, Makoto Takahashi, Koichi Shinkoda

**Affiliations:** 1 Graduate School of Biomedical and Health Sciences, Hiroshima University, 2-3 Kasumi 1-chome, Minami-ku, Hiroshima, 734-8553 Japan; 2 Department of Biomechanics, Graduate School of Biomedical and Health Sciences, Hiroshima University, 2-3 Kasumi 1-chome, Minami-ku, Hiroshima, 734-8553 Japan; 3 The Center for Advanced Practice and Research of Rehabilitation, Graduate School of Biomedical and Health Sciences, Hiroshima University, 2-3 Kasumi 1-chome, Minami-ku, Hiroshima, 734-8553 Japan

## Correction

After the publication of this work [1] an error was noticed in Table 3. In the column ‘Elderly’, in the last row it states 98 ± 11.87** however the correct value should be: 34.98 ± 11.87**.

The original article has been corrected.
